# A systematic genetic screen identifies essential factors involved in nuclear size control

**DOI:** 10.1371/journal.pgen.1007929

**Published:** 2019-02-13

**Authors:** Helena Cantwell, Paul Nurse

**Affiliations:** 1 Cell Cycle Laboratory, The Francis Crick Institute, London, United Kingdom; 2 Laboratory of Yeast Genetics and Cell Biology, Rockefeller University, New York, United States of America; Stanford University, UNITED STATES

## Abstract

Nuclear size correlates with cell size, but the mechanism by which this scaling is achieved is not known. Here we screen fission yeast gene deletion mutants to identify essential factors involved in this process. Our screen has identified 25 essential factors that alter nuclear size, and our analysis has implicated RNA processing and LINC complexes in nuclear size control. This study has revealed lower and more extreme higher nuclear size phenotypes and has identified global cellular processes and specific structural nuclear components important for nuclear size control.

## Author summary

As cells grow and divide, the size of the nucleus is generally maintained as a fixed proportion of cell size. The mechanism by which this nuclear/cytoplasmic ratio is maintained is unclear. Previous studies have suggested that essential gene products may be important for nuclear size control. Therefore, we have exploited the genetic tractability of fission yeast to carry out a systematic genetic screen of deleted essential genes to identify those with aberrant nuclear size phenotypes. Our study has revealed 25 novel genes that influence nuclear size and our bioinformatic analyses have implicated both RNA processing and protein complexes connecting nuclear chromatin to the cytoskeleton in nuclear size control. Our work sheds light on the biological processes that contribute to nuclear size control in fission yeast contributing to our mechanistic understanding of nuclear scaling, a biological phenomenon that is conserved through evolution.

## Introduction

Study of sea urchin embryos led Hertwig more than a century ago to the Kern-Plasma-relation theory, which proposes that the ratio between nuclear size and cell size is constant [1]. A constant ratio between nuclear and cell size has since been reported in many cell types from unicellular yeasts and Tetrahymena to cells of multicellular animals and plants [2–11]. In multicellular organisms, the nucleocytoplasmic ratio varies between cell types, but is generally restricted to a narrow range for cells of each type [10].

There are several lines of evidence that nuclear volume is determined by cell volume, or a factor related to it, and not directly by DNA content. Nuclear volume correlates with cell volume across a wide range of cell sizes in both budding [6] and fission [7] yeasts. In fission yeast, genetic mutations and nutritional states were used to generate cells displaying a 35-fold range of cell volumes; the nuclear volume to cell volume (N/C) ratio was observed to be constant across this range [7]. In small scale pilot experiments, nuclear volume increased steadily with cell volume during the cell cycle, maintaining a constant N/C ratio throughout interphase; no sudden increase in nuclear size accompanied DNA replication in S phase [6, 7]. Gradual nuclear growth during interphase was also observed in HeLa cells [12]. These data indicate that nuclear volume is not directly determined by DNA content. Even a 16-fold increase in DNA content was not sufficient to alter the N/C ratio of fission yeast cells [7]. Furthermore, in situations where DNA content remained constant, nuclear volume responded to changes in cell volume; transfer of the nucleus of a hen erythrocyte into the cytoplasm of a larger HeLa cell resulted in an increase in nuclear volume [13], as did transfer of HeLa cell nuclei into the cytoplasm of a larger *Xenopus* oocyte [14]. Nuclear volume was observed to increase concomitantly with cell volume following treatment of murine hepatocytes with c-Myc [15], and to scale with cell volume during the reductive divisions of post-16 cell stage *C*. *elegans* embryogenesis [9]. However, the mechanism by which cell volume influences nuclear volume has not been established. Studies of centrifuged sea snail embryos and multinucleate yeast cells have demonstrated that diffusible cytoplasmic factors, rather than structural constraints of cell volume, influence nuclear size [5, 7]. Nuclear import of lamins has been implicated in nuclear size control in *Xenopus* and human cells [8, 16], but yeasts, which lack lamins, also display nuclear scaling [6, 7], so there must be other factors central to nuclear size control.

The fission yeast *Schizosaccharomyces pombe* is genetically tractable, simply shaped and undergoes a closed mitosis, making it a useful system in which to probe the mechanism of nuclear size control *in vivo*. A screen of non-essential *S*. *pombe* gene deletion mutants implicated both bulk nucleocytoplasmic transport and regulation of lipid biosynthesis in nuclear size control [17]. However, no mutants with a low N/C ratio phenotype were identified, leading us to hypothesise that such a phenotype may be sufficiently deleterious to be lethal, and therefore key nuclear size control regulators may be encoded by essential genes [17]. Therefore, to identify novel candidate factors required for the control of nuclear size during interphase, we have carried out a genetic screen of *S*. *pombe* essential gene deletion mutants for those exhibiting aberrant N/C ratios. This is the first reported systematic study of the role of products of essential genes in nuclear size control and has uncovered both low N/C ratio and severely aberrant N/C ratio deletion phenotypes.

## Results

### Phenotypic screen of *S*. *pombe* essential gene deletion mutants identified 25 nuclear size mutants

**Fig 1:**
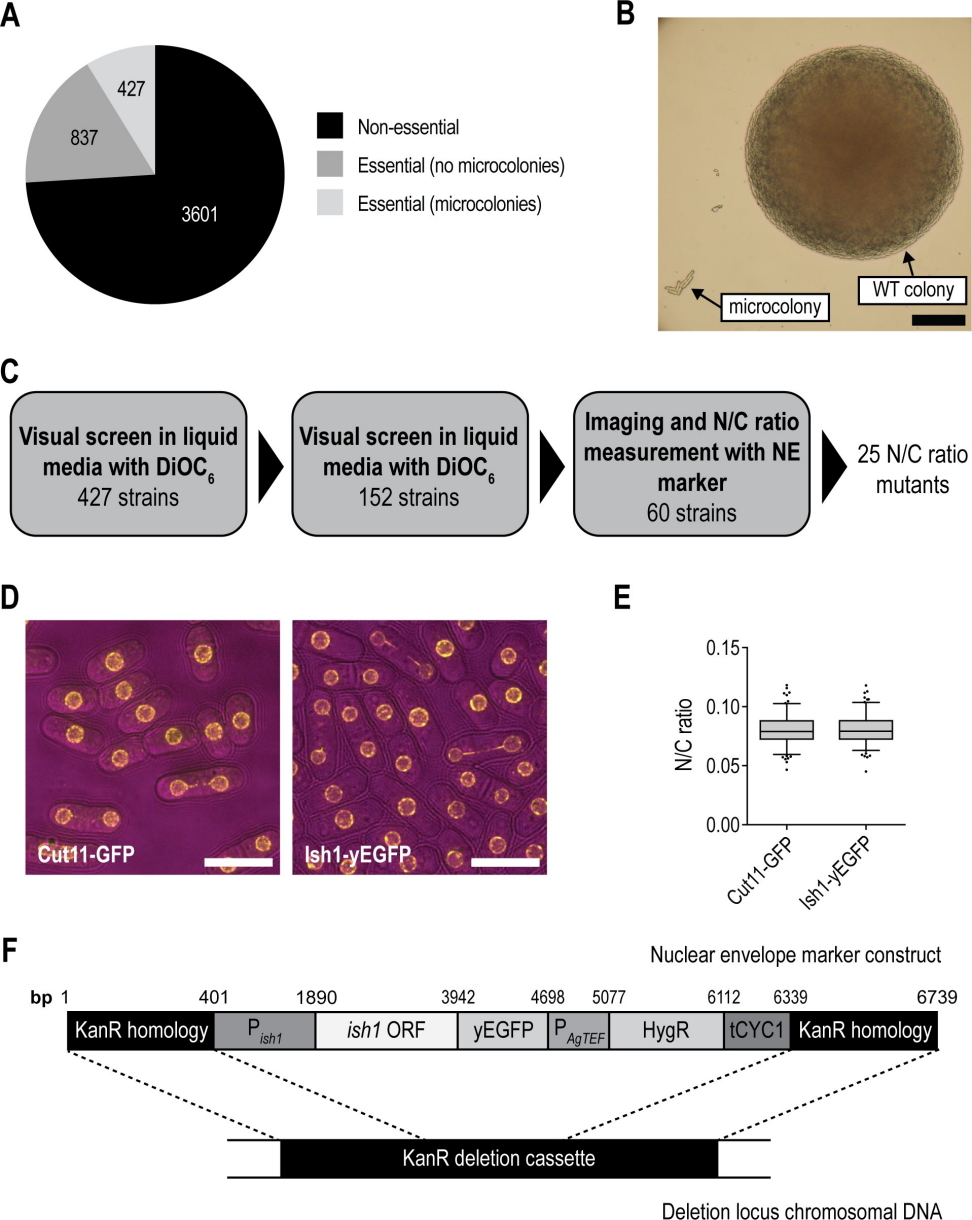
Microcolony forming essential gene deletion mutants were screened by insertion of a nuclear envelope marker. (A) Pie chart illustrating stratification of *S*. *pombe* gene deletion collection into non-essential and essential gene deletion mutants. Essential gene deletion mutants are then categorised by ability to form microcolonies [18, 19]. (B) Representative image of wild type (WT) colony and microcolony grown on solid YE4S agar from spores from sporulated heterozygous deletion mutant diploid (40 h, 32°C). Scale bar: 100 μm. (C) Schematic of the three stage screen. Two visual screens in liquid media using the lipophilic dye DiOC_6_ identified 60 potential N/C ratio mutant candidates. Imaging and measurement of N/C ratio of strains containing nuclear envelope (NE) marker Ish1-yEGFP led to the identification of 25 N/C ratio mutant candidates. (D) Wild type haploid cells with nuclear envelopes marked with Cut11-GFP or Ish1-yEGFP as indicated. Brightfield (magenta), GFP (yellow). Scale bar: 10 μm. (E) N/C ratio of cell populations described in (D). n = 150 cells per condition. Box delimited by 25^th^ percentile, median and 75^th^ percentile. Whiskers represent 10^th^ and 90^th^ percentiles. Data points outside this range marked as dots. Unpaired student’s t-test carried out (p = 0.6871). (F) Schematic of nuclear envelope marker construct. Kanamycin resistance marker (KanR) homologous sequence flanks the *ish1* open reading frame (*ish1* ORF) and upstream sequence (P_*ish1*_), yEGFP coding sequence (yEGFP) and hygromycin resistance marker (HygR) regulated by the AgTEF promoter (P_*AgTEF*_) and CYC1 terminator (tCYC1).

**Fig 2:**
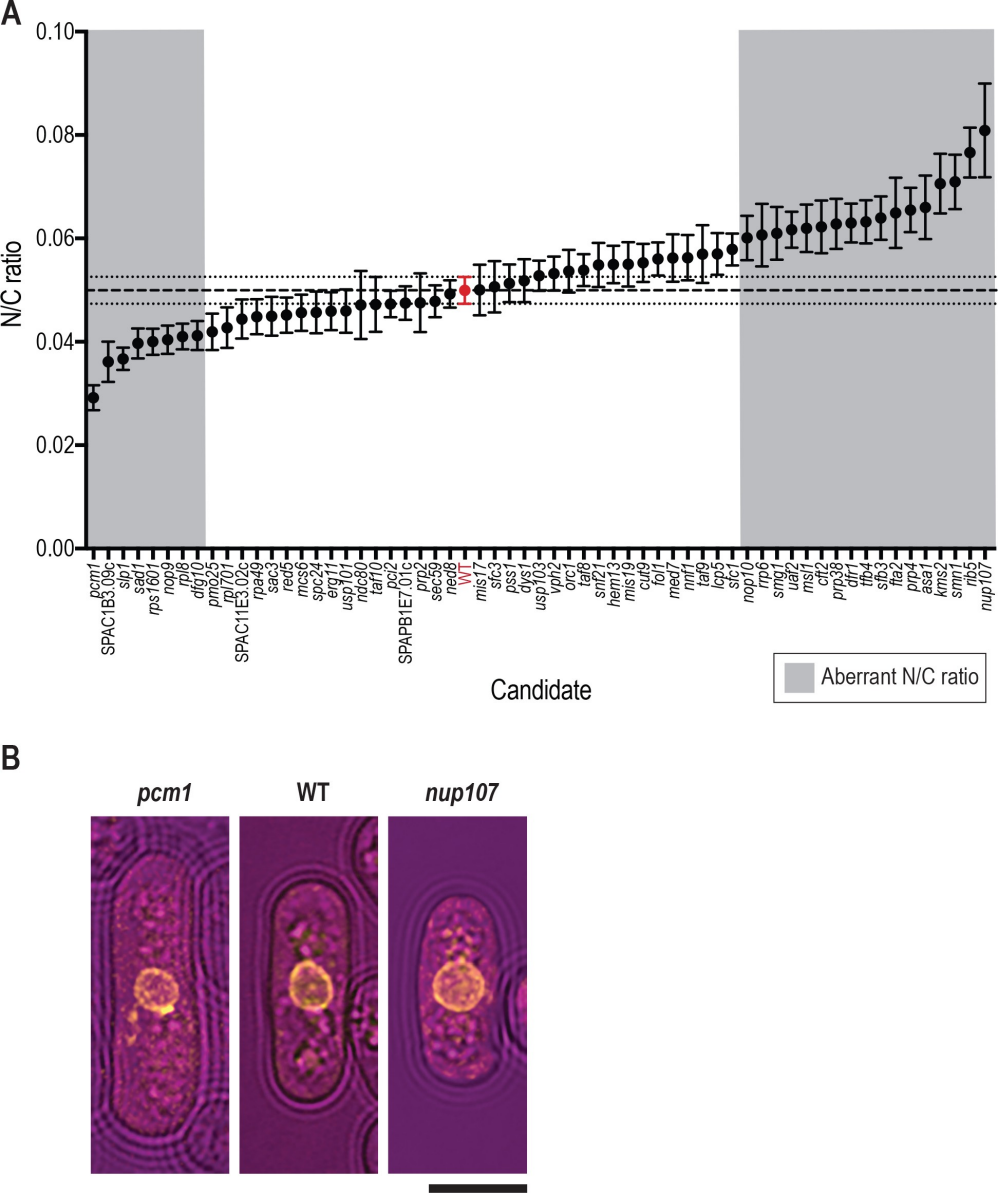
25 gene deletion mutants with aberrant N/C ratios identified by the genetic screen. (A) N/C ratio of wild type cells (red) and 60 gene deletion mutants in the third screen with nuclear envelope marker Ish1-yEGFP integrated in the deletion cassette. Mean plotted for each strain (n = 50 cells per strain). Error bars represent 95% confidence intervals. Dashed line represents wild type cell population mean. Dotted lines represent wild type 95% confidence intervals. Wild type strain used is the haploid derived from 5300 control strain (red). Aberrant N/C ratio candidates (grey background) were identified as described in Materials and Methods. (B) Images of an example cell for wild type (WT) and strains carrying deletions in *pcm1* and *nup107*. Brightfield (magenta), Ish1-yEGFP (yellow). Scale bar: 5 μm.

We screened essential gene deletion mutants from a near genome-wide heterozygous gene deletion collection that represents 99% of *S*. *pombe* open reading frames [18, 19]. Following sporulation of the heterozygous diploids, haploid deletion mutants of 427 of the *S*. *pombe* essential genes divide a limited number of times following germination to form microcolonies of 4–20 cells (Fig 1A and 1B) allowing their N/C ratio phenotype to be assessed. In three stages, we visually screened these haploid microcolony-forming essential gene deletion mutants for those that exhibit aberrant N/C ratios (Fig 1C, Materials and Methods). Nuclear size was monitored, initially using a lipophilic dye and subsequently the nuclear envelope (NE) marker Ish1-yEGFP (Fig 1D, 1E and 1F). In the third stage of the screen cell size and nuclear size were measured, using brightfield and Ish1-yEGFP fluorescence images respectively, for 50 cells of each strain, and the N/C ratio was calculated (Materials and Methods). This yielded reliable estimates of the N/C ratio of each strain population; for example the wild type population mean was 0.05 ± 0.0026 (95% confidence interval) (Fig 2A). Note that the introduction of an additional copy of Ish1 into cells for tertiary screening influences the N/C ratio (see Materials and Methods). This methodology provides an approach to systematically probe essential gene deletion phenotypes. However, it should be noted that the cells will eventually die as the microcolonies do not proceed to form colonies, and so cells may not be in a steady state equilibrium. The eventual loss of viability in microcolony cells could be due to the reduced concentration of maternal gene product diluted by sequential cell divisions, or to an accumulating stochastic loss of viability due to the gene deletion.

**Fig 3:**
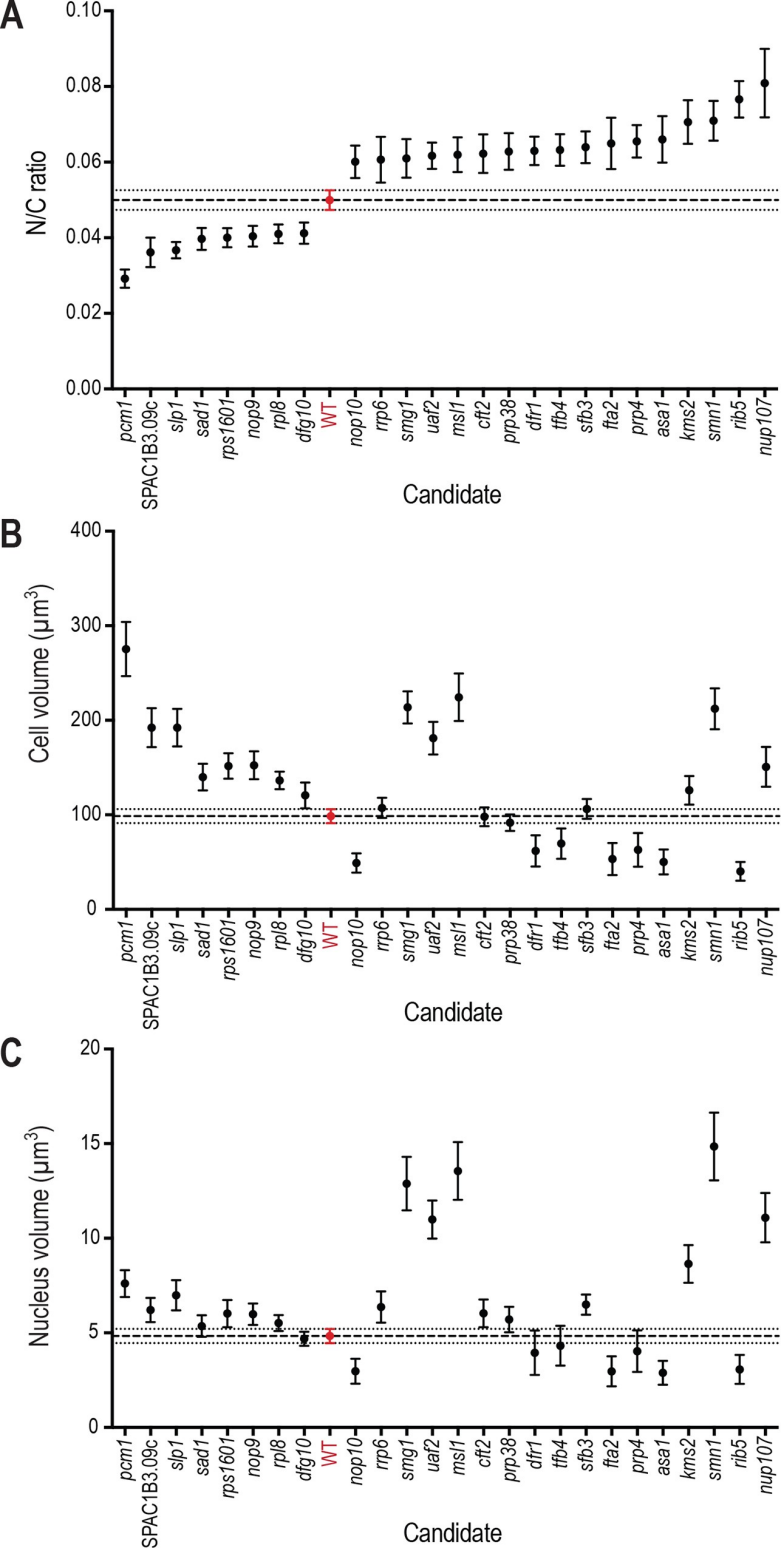
N/C ratio, cell volume and nucleus volume measurements of aberrant N/C ratio mutants. (A-C) N/C ratio (A), Cell volume (B) and Nucleus volume (C) of wild type cells (red) and 25 gene deletion mutants in the third screen with nuclear envelope marker Ish1-yEGFP integrated in the deletion cassette. Mean plotted for each strain (n = 50 cells per strain). Error bars represent 95% confidence intervals. Dashed line represents wild type cell population mean. Dotted lines represent wild type 95% confidence intervals. Wild type strain used is the haploid derived from 5300 control strain (red).

**Fig 4:**
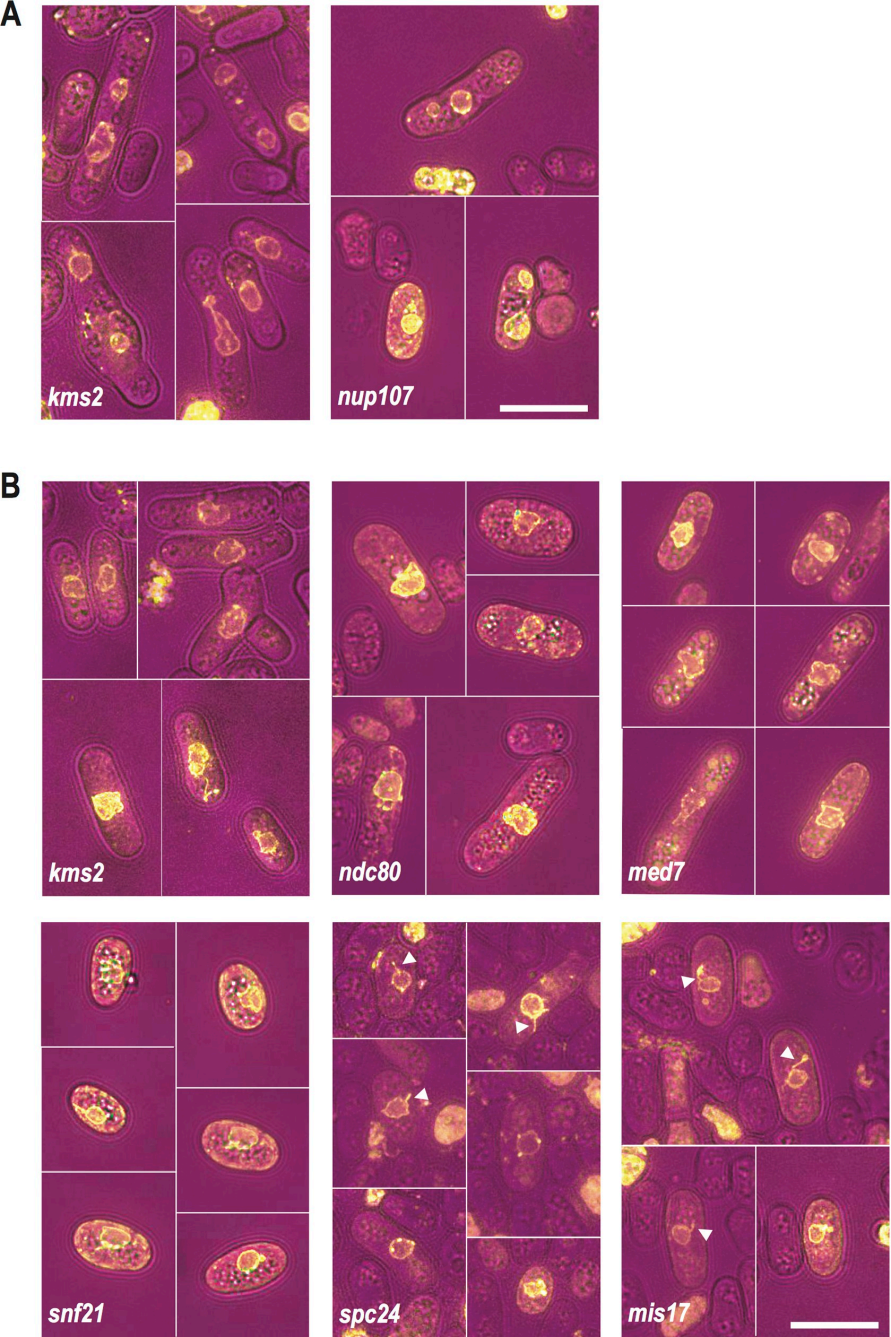
Deletion mutants displaying nuclear shape defects or asymmetric nuclear division. (A) Images of asymmetric nuclear division in strains with genes indicated deleted. Brightfield (magenta), Ish1-yEGFP (yellow). Scale bar: 10 μm. (B) Representative images of strains carrying deletions in the genes indicated identified as having nuclear shape defects during screen. Nuclear tethers indicative of defective microtubule-spindle pole body attachment [22] were observed in *spc24Δ* and *mis17Δ* cells and are labelled by white triangles. Brightfield (magenta), Ish1-yEGFP (yellow). Scale bar: 10 μm.

Of the 60 mutants screened with Ish1-yEGFP, 25 were identified as aberrant N/C ratio candidates (Fig 2A, S1 Table). Our screen revealed both low N/C ratio phenotypes, and high N/C ratio phenotypes more extreme than those previously identified. Seventeen of the candidates identified displayed an N/C ratio higher, and 8 lower, than wild type cells. Representative images of the most extreme high and low N/C ratio candidates and wild type cells are shown in Fig 2B, and of all N/C ratio mutant candidates in S1 Appendix. Cell length, cell width, cell volume and nucleus volume measurements for all 25 candidates, and for the wild type control, are shown in Table 1, and cell volume, nucleus volume and N/C ratio measurements are displayed in Fig 3. The most extreme low N/C ratio observed was 42% smaller than the wild type value (*pcm1Δ*, lacking the gene encoding the P-TEFb-cap methyltransferase) and the most extreme high N/C ratio was 62% greater than wild type (*nup107Δ*, lacking the gene encoding nucleoporin Nup107). In the *pcm1Δ* mutant, average cell volume was larger than that of wild type cells, but both the enlarged cells and the smallest cells in the population (which had approximately wild type cell volumes) displayed diminished N/C ratios. Two mutants with altered N/C ratio were observed to undergo asymmetric nuclear division (Fig 4A). These were *kms2Δ* (lacking the gene encoding linker of nucleoskeleton and cytoskeleton (LINC) complex component Kms2), for which mitotic defects have previously been described [20], and *nup107Δ*. Mitotic defects have been reported for mutants of other components of the Nup107-120 complex [21]. In addition to N/C ratio changes, we also identified six deletion mutants with nuclear shape defects; for example nuclear tethers thought to be indicative of defective microtubule-spindle pole body attachment [22] were observed in *spc24Δ* and *mis17Δ* cells which carry deletions of kinetochore components (Fig 4B).

**Table 1:** N/C ratio mutants identified by the genetic screen.

Gene deleted	Cell length (μm)	Cell width (μm)	Cell volume (μm^3^)	Nucleus volume (μm^3^)	N/C ratio
*pcm1*	16.0 ± 4.9	4.9 ± 0.5	275.3 ± 100.8	7.6 ± 2.5	0.029 ± 0.009
SPAC1B3.09c	9.9 ± 1.9	5.4 ± 0.6	192.2 ± 72.1	6.2 ± 2.3	0.035 ± 0.013
*slp1*	10.9 ± 2.0	5.1 ± 0.6	192.3 ± 69.8	7.0 ± 2.8	0.037 ± 0.008
*sad1*	9.9 ± 2.2	4.5 ± 0.5	139.9 ± 49.8	5.4 ± 2.0	0.040 ± 0.010
*rps1601*	9.9 ± 1.8	4.8 ± 0.5	151.7 ± 47.3	6.0 ± 2.5	0.040 ± 0.009
*nop9*	10.5 ± 2.2	4.6 ± 0.5	152.4 ± 52.0	6.0 ± 2.0	0.040 ± 0.010
*rpl8*	9.7 ± 1.7	4.6 ± 0.4	136.4 ± 32.9	5.5 ± 1.5	0.041 ± 0.009
*dfg10*	8.2 ± 1.6	4.8 ± 0.5	120.6 ± 47.9	4.7 ± 1.3	0.041 ± 0.010
WT	8.3 ± 1.4	4.2 ± 0.3	98.6 ± 26.1	4.8 ± 1.3	0.050 ± 0.009
*nop10*	7.1 ± 2.0	3.0 ± 0.8	49.1 ± 36.0	3.0 ± 2.3	0.060 ± 0.015
*rrp6*	8.9 ± 2.1	4.2 ± 0.5	107.3 ± 37.6	6.4 ± 2.9	0.061 ± 0.021
*smg1*	12.7 ± 3.3	5.0 ± 0.5	213.7 ± 59.7	12.9 ± 5.0	0.061 ± 0.018
*uaf2*	10.8 ± 2.2	5.0 ± 0.4	181.2 ± 60.7	11.0 ± 3.5	0.062 ± 0.012
*msl1*	13.3 ± 4.0	4.9 ± 0.6	224.4 ± 88.6	13.6 ± 5.4	0.062 ± 0.016
*cft2*	7.9 ± 2.1	4.4 ± 0.4	98.0 ± 35.0	6.0 ± 2.6	0.062 ± 0.018
*prp38*	9.4 ± 2.0	3.8 ± 0.5	91.7 ± 30.6	5.7 ± 2.4	0.063 ± 0.017
*dfr1*	6.8 ± 2.7	3.3 ± 0.9	61.8 ± 57.9	3.9 ± 4.1	0.063 ± 0.013
*tfb4*	8.5 ± 2.9	3.1 ± 0.8	69.6 ± 56.4	4.3 ± 3.7	0.063 ± 0.015
*sfb3*	8.6 ± 1.9	4.3 ± 0.5	106.2 ± 37.0	6.5 ± 1.9	0.064 ± 0.015
*fta2*	6.3 ± 2.5	3.1 ± 0.9	53.2 ± 59.9	3.0 ± 2.8	0.065 ± 0.024
*asa1*	5.7 ± 1.8	3.3 ± 1.0	50.2 ± 46.4	2.9 ± 2.2	0.066 ± 0.022
*prp4*	7.3 ± 2.4	3.4 ± 1.0	68.4 ± 65.9	4.4 ± 4.0	0.067 ± 0.014
*kms2*	9.8 ± 2.9	4.4 ± 0.5	125.9 ± 53.2	8.6 ± 3.5	0.071 ± 0.020
*smn1*	13.5 ± 3.9	4.8 ± 0.6	212.2 ± 76.2	14.8 ± 6.3	0.071 ± 0.019
*rib5*	5.9 ± 1.9	2.9 ± 0.9	40.2 ± 34.8	3.1 ± 2.7	0.077 ± 0.017
*nup107*	9.5 ± 2.4	4.8 ± 0.7	150.8 ± 73.8	11.1 ± 4.6	0.081 ± 0.032

Cell length, cell width, cell volume, nucleus volume and N/C ratio measurements for the 25 N/C ratio mutant candidates identified in screen and the wild type control strain (WT). Mean ± standard deviation. n = 50 cells per strain.

We assessed the relationships between cellular and nuclear volume and N/C ratio in our screen candidates (S1A and S1B Fig). Mean nuclear volumes ranged from 2.22 μm^3^ to 14.9 μm^3^, compared to the wild type of 4.83 μm^3^. Mean cellular volumes were mostly between 90 μm^3^ and 200 μm^3^, with extremes at 40.2 μm^3^ and 275 μm^3^, compared to 98.6 μm^3^ in wild type. All 8 low N/C ratio mutants displayed mean cellular volumes higher than that of the wild type strain, ranging from 121 μm^3^ to 275 μm^3^. The 17 high N/C ratio mutants displayed mean cellular volumes both greater and smaller than that of wild type, ranging from 40.2 μm^3^ to 224 μm^3^. We analysed the cellular volume and N/C ratio measurements for individual cells within the populations. For all except two of the high N/C ratio and low N/C ratio strains, there was a weak negative correlation between cellular volume and N/C ratio, as is the case in wild type cells, however the gradient of the regression line of this did not correlate with mean N/C ratio of the population (S1C Fig). When only cells with cellular volumes within two standard deviations of the wild type mean were considered, despite lower n values, 21 of the 25 N/C ratio mutants identified by our screen still displayed significantly aberrant N/C ratios (S2 Table). Therefore, the N/C ratio mutants we identified are not a consequence of aberrantly sized cells with wild type sized nuclei.

If vacuolar size was altered in N/C ratio mutants then cytoplasmic volume may be less directly related to cell volume than in wild type cells, but no changes in the vacuoles of the mutants were observed. The vacuoles in wild type cells and the most extreme N/C ratio mutants, visualised using the vacuole staining dye FM 4–64, are shown in S2 Fig. No differences were observed in the N/C ratio mutants compared with wild type cells.

### Nuclear size mutants are enriched for RNA processing factors and nucleus-localised proteins

To determine whether specific categories of genes are enriched in the genes deleted in nuclear size mutants identified in *S*. *pombe*, we carried out gene ontology (GO) enrichment analysis (Table 2) on both those identified in this study (Table 1) and also our previous non-essential genes screen [17]. Relative to all genes screened, the 33 nuclear size genes are enriched for genes in the GO biological process (GOBP) categories mRNA metabolic process, mRNA processing, RNA processing and gene expression. The greatest enrichment (8.64-fold) was observed for mRNA processing genes. The GO cellular component (GOCC) categories LINC complex, Nem1-Spo7 phosphatase complex, intracellular ribonucleoprotein complex, ribonucleoprotein complex, nuclear part, macromolecular complex and protein complex categories were also enriched.

**Table 2:** GO annotation categories enriched in nuclear size mutants.

GO category		Nuclear size mutants		Background		Fold enrichment	p value
		Frequency	%	Frequency	%		
GOBP	mRNA processing	8/33	24.2	96/3377	2.8	8.6	0.00057
	mRNA metabolic process	10/33	30.3	160/3377	4.7	6.5	0.00036
	RNA processing	12/33	36.4	258/3377	7.6	4.8	0.00059
	gene expression	20/33	60.6	849/3377	25.1	2.4	0.00355
GOCC	LINC complex	2/33	6.1	2/3377	0.1	61	0.00787
	Nem1-Spo7 phosphatase complex	2/33	6.1	2/3377	0.1	61	0.00787
	intracellular ribonucleoprotein complex	10/33	30.3	260/3377	7.7	3.9	0.00988
	ribonucleoprotein complex	10/33	30.3	260/3377	7.7	3.9	0.00988
	nuclear part	20/33	60.6	779/3377	23.1	2.6	0.00031
	protein complex	16/33	48.5	627/3377	18.6	2.6	0.00705
	macromolecular complex	27/33	81.8	1082/3377	32.0	2.6	3.87 x10^-7^

GO annotation categories enriched in genes deleted in 33 nuclear size mutants relative to background population of all essential and non-essential genes screened (p ≤ 0.01) are shown. Adjusted p values calculated using the Bonferroni multiple hypothesis testing correction method. GO biological process (GOBP) and GO cellular component (GOCC) are shown. No GO molecular function (GOMF) categories were significantly enriched. Enrichment analysis performed using GO::TermFinder [23] with PomBase annotations [24].

### Network analysis highlights interactions between genes with nuclear size mutant deletion phenotypes

**Fig 5:**
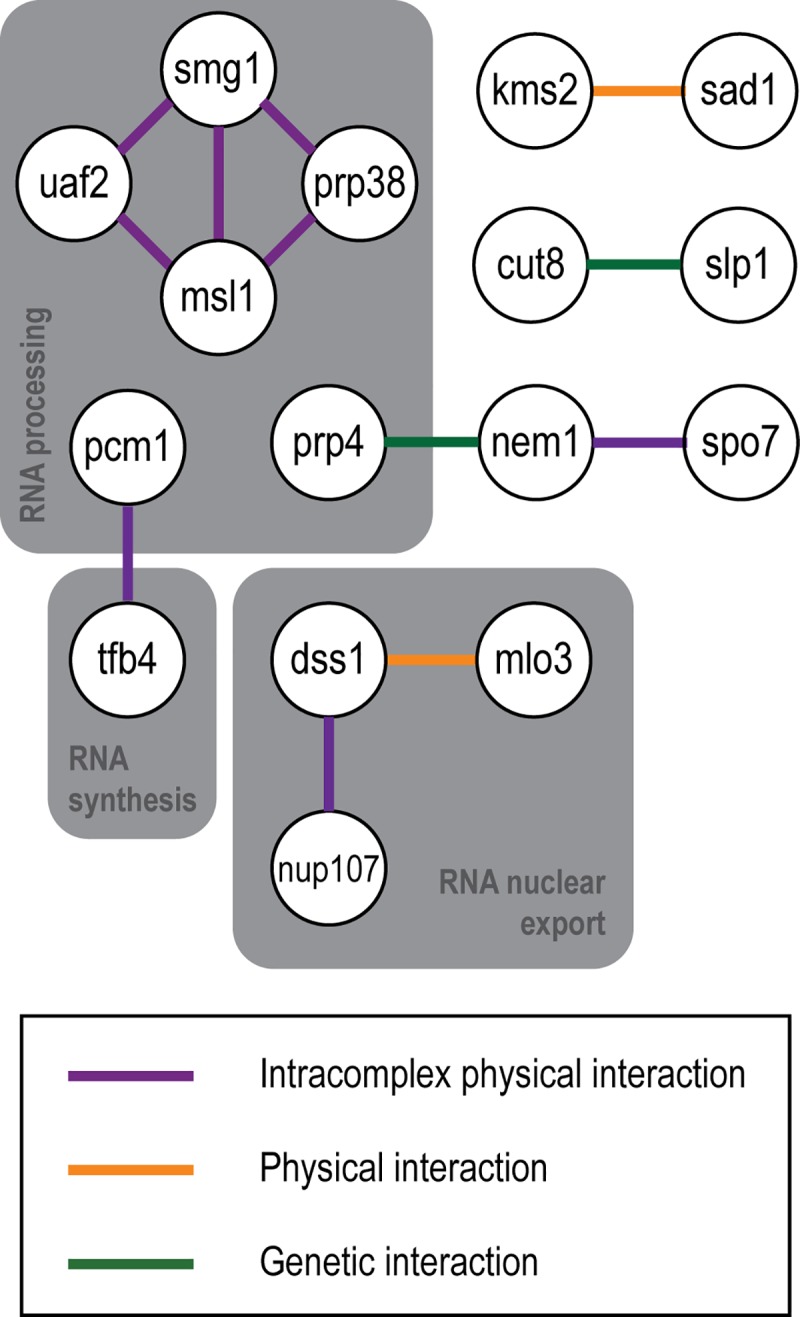
Network analysis displays reported interactions between nuclear size mutant genes. Network analysis carried out using esyN software [25] with PomBase [24] and BioGRID [36] curated interactions. Interacting candidate factors with roles in RNA processing, synthesis and nuclear export indicated.

Gene interactions were identified by network analysis [25] (Fig 5). Two components of the Dss1-Mlo3 mRNA export complex and two components of the Nem1-Spo7 phosphatase involved in lipid biogenesis, were previously identified [17]. Also identified were another interactor of Nem1, protein kinase Prp4, which is reported to negatively genetically interact with Nem1, and Nup107, a nucleoporin reported to physically interact with Dss1.

Two components of the LINC complex, Kms2 and Sad1, Tfb4, of the TFIIH complex, and Pcm1, of the P-TEFb-cap methyltransferase complex, were identified. These latter proteins both have roles in transcription, acting as part of the DNA-directed RNA polymerase II holoenzyme. Deletion mutants of the nuclear proteasome tether Cut8 and anaphase promoting complex (APC) coactivator Slp1 both have aberrant nuclear size and interact genetically, displaying synthetic lethality [26]. Four spliceosomal proteins Smn1, Uaf2, Smg1, Prp38 and Msl1 were also identified.

### Activity of the P-TEFb complex influences nuclear size

**Fig 6:**
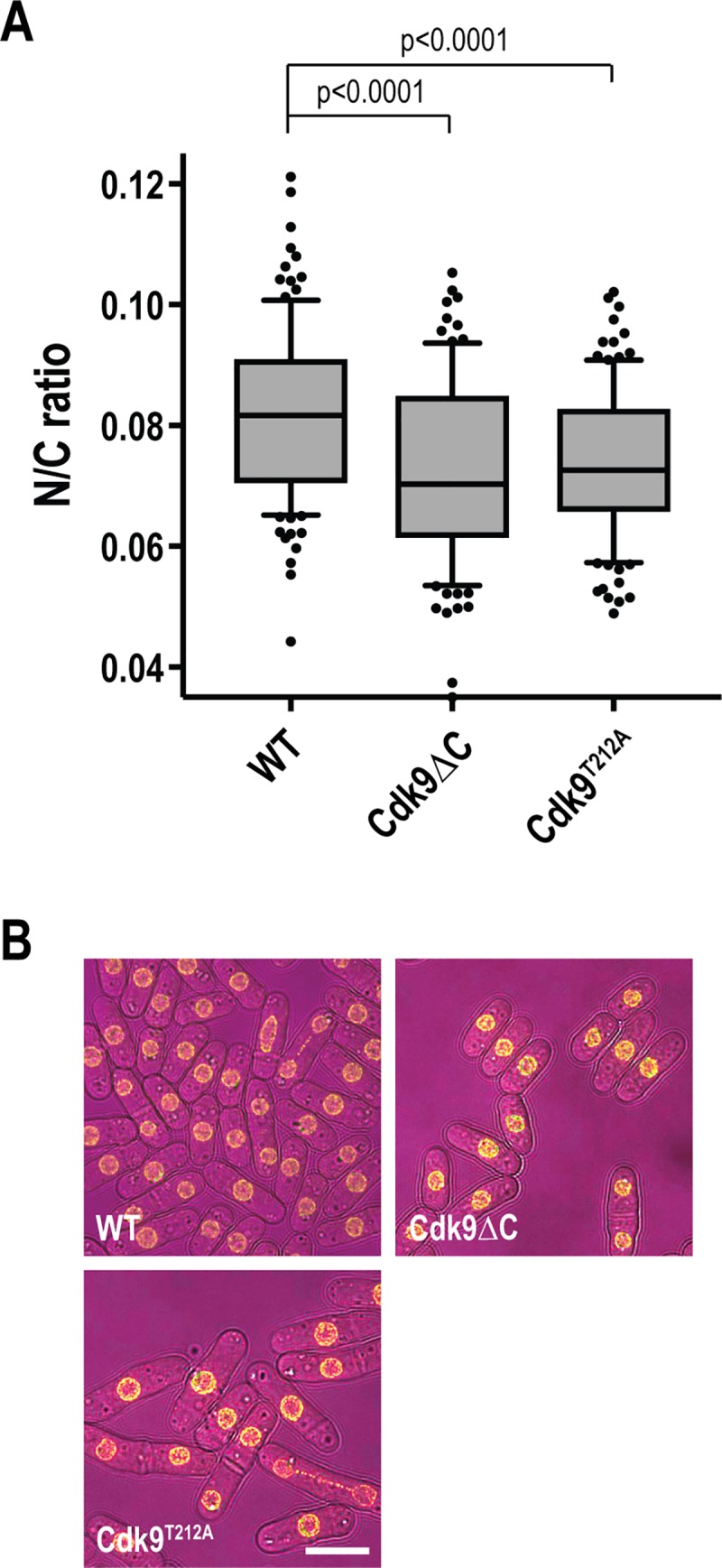
Cdk9 mutants display low N/C ratio phenotypes. (A) N/C ratio of wild type (WT) and Cdk9 C-terminal truncation (Cdk9*Δ*C) and T-loop (Cdk9^T212A^) mutant cells grown at 25°C. n ≥ 100 cells per condition. Box delimited by 25^th^ percentile, median and 75^th^ percentile. Whiskers represent 10^th^ and 90^th^ percentiles. Data points outside this range marked as dots. Unpaired student’s t-tests with Welch’s correction carried out to determine significance. Cdk9*Δ*C retains kinase activity but loses the ability to bind to Pcm1, reducing recruitment of both Cdk9 and Pcm1 to chromatin [28]. In *cdk9*^*T212A*^ cells Cdk9 activation by CDK-activating kinase is perturbed [28]. (B) Representative images of wild type (WT) and Cdk9 C-terminal truncation (Cdk9*Δ*C) and T-loop (Cdk9^T212A^) mutants cells grown at 25°C. Brightfield (magenta), Cut11-GFP (yellow). Scale bar: 10 μm.

The most extreme low N/C ratio mutant was a mutant with a deletion of the gene encoding Pcm1, displaying an N/C ratio 58% of the wild type value. The methyltransferase Pcm1 forms the P-TEFb-methyltransferase complex, with cyclin dependent kinase (CDK) Cdk9 and cyclin Pch1, required for 7-methylguanosine capping of mRNA [27]. A C-terminal truncation of Cdk9 (Cdk9*Δ*C) retains kinase activity but loses the ability to bind to Pcm1; lack of this interaction reduces recruitment of both Cdk9 and Pcm1 to chromatin [28]. We hypothesised that if Pcm1’s role in the P-TEFb-methyltransferase complex is required for appropriate nuclear size control, then Cdk9*Δ*C cells should, like *pcm1Δ* cells, display aberrant nuclear size. We observed an N/C ratio significantly lower than that of wild type cells in Cdk9*Δ*C cells (Fig 6A and 6B). A Cdk9 T loop mutant, Cdk9^T212A^, in which Cdk9 activation by CDK-activating kinase (CAK) is perturbed [28] also displayed an N/C ratio lower than wild type cells (Fig 6A and 6B), suggesting that the recruitment of Cdk9 to chromatin by Pcm1 and Cdk9 activity are important for nuclear size control.

## Discussion

Here, we describe a systematic genetic screen of *S*. *pombe* essential genes for those with deletion mutants that display interphase nuclear size defects and so encode factors that may have roles in nuclear size control. This screen identified 25 potential nuclear size mutants, including 8 with low N/C ratios. This is the first report of mutants displaying low interphase N/C ratios. A previously reported genetic screen of *S*. *pombe* non-essential genes identified no low N/C ratios [17]. This essential genes screen also identified mutants with high N/C ratio phenotypes, and mutants with altered nuclear shape.

Nuclear size mutants were enriched for genes encoding products with roles in RNA processing, and gene expression more generally. As processing of mRNAs into mRNPs is required for their nuclear export [29], it is possible that perturbed RNA processing could lead to the nuclear accumulation of defective mRNA transcripts, which in turn could lead to N/C ratio alteration by bulk effects analogous to those observed in the previously reported mRNA export defective mutant *rae1-167* that displays an enlarged N/C ratio [17]. Alternatively, accumulation of the proteins themselves may influence the N/C ratio; proteins with these functions are often found as part of large complexes comprised of many protein and RNA components. Perturbing the stoichiometry or localisation of a large complex of this kind could influence nucleocytoplasmic transport by affecting the distribution of transport factors between the nucleus and cytoplasm and thus preventing the nucleocytoplasmic transport of other proteins. A further possibility is that altering gene expression or mRNA processing influences expression level of specific RNAs and proteins important for N/C ratio control. In mutants with low N/C ratio, the level of expression of genes encoding proteins required for lipid synthesis may be reduced, limiting nuclear size.

Nuclear size mutant gene products were enriched for proteins localised to the nucleus and ribonucleoprotein complexes. The largest group of interactors was a group of spliceosomal proteins suggesting that this ribonucleoprotein complex, or the appropriate regulation of its synthesis or nucleocytoplasmic transport, is important for nuclear size control. Perturbation of mRNA synthesis or mRNA processing will influence the number of ribosomes which could be related to the monitoring of cell size and thus alter the N/C ratio. The strain identified with the lowest N/C ratio was *pcm1Δ*. Pcm1 forms part of the P-TEFb-methyltransferase complex, another example of a ribonucleoprotein, which is required for 5’ capping of mRNA. Mutants of Cdk9, another component of this complex, also displayed a low N/C ratio suggesting that activity of the P-TEFb-methyltransferase complex is also important for nuclear size control.

Both GO enrichment and network analysis implicated the LINC complex and the Nem1-Spo7 phosphatase complex in nuclear size control. The role of the Nem1-Spo7 complex in the regulation of lipid synthesis for nuclear size control has been described previously [17]. LINC complexes are conserved protein complexes that bridge the nuclear envelope, connecting nuclear chromatin to the cytoskeleton [30]. In *S*. *pombe*, the KASH domain containing integral outer nuclear membrane protein Kms2 and the SUN domain containing integral inner nuclear membrane protein Sad1 form these bridging complexes [20]. Both *kms2Δ* and *sad1Δ* were identified as N/C ratio mutant candidates by genetic screening. It has been suggested that LINC complexes buffer forces on the nuclear envelope preserving nuclear morphology [20], so they may constrain nuclear expansion. This possibility is supported by data from mammalian cells which contain multiple KASH domain containing proteins. Four of them are nesprins consisting of a N-terminal actin binding domain (ABD) separated from a C-terminal transmembrane KASH domain by an extended domain of spectrin repeats, and these are thought to form a filamentous network on the cytoplasmic face of the nuclear envelope [31]. In HaCaT cells, disruption of these interchain interactions by overexpression of the ABD of Nesprin-2 increased nuclear size and expression of reduced length Nesprin-2 decreased nuclear size, demonstrating that the interactions between KASH domain proteins are important for nuclear size control in these cells [31].

Though not the main focus of our study, the screening process also identified deletion mutants with previously unreported nuclear shape defects and asymmetric nuclear division phenotypes. Six mutants with nuclear shape defects were identified; among these were three kinetochore proteins, Ndc80, Spc24 and Mis17, and the LINC complex protein Kms2, supporting a proposed model where LINC complexes buffer inward forces from microtubules on the nuclear envelope [20]. Two mutants were observed to display asymmetric nuclear division, *kms2Δ*, in which mitotic defects were previously described [20], and a strain carrying a deletion of the gene encoding the nucleoporin Nup107. Mitotic defects have previously been reported in mutants of other components of the Nup107-120 complex, as have defects in Ran nuclear transport [21]; either of these perturbations could lead to asymmetric nuclear division.

This genetic screen of fission yeast has uncovered low and more extreme high N/C ratio phenotypes, and revealed novel roles for essential factors in nuclear size control. Bioinformatic analyses have implicated RNA processing and LINC complexes in the control. This suggests that both the regulation of global cellular processes, balancing levels of nucleoplasmic transcription and cytoplasmic translation, and regulation of the levels of specific structural components of the nucleus are important for nuclear size control.

## Materials and methods

### Strains and growth conditions

*S*. *pombe* media and methods used were as described previously [32]; cells were grown in YE4S unless otherwise indicated. *S*. *pombe* strains used in this study are listed in S3 Table. Gene tagging was performed using PCR-based methods described previously [33] and verified by colony PCR. The following alleles were previously reported: *cdk9ΔC* [28] and *cdk9*^*T212A*^ [28].

### Systematic genetic screen

427 strains from a near genome-wide heterozygous gene deletion collection carrying deletions of essential genes and reported to form haploid microcolonies [18, 19] were screened in a primary screen for aberrant N/C ratio mutants.

Heterozygous diploids were patched from glycerol stocks onto YE agar + 250 mg/L uracil + 250 mg/L leucine + 100 μg/ml G418 and incubated at 32°C for 3 days then grown into stationary phase in 200 μl YE + 250 mg/L uracil + 250 mg/L leucine + 250 mg/L adenine liquid media in 96-well plates. Cells were then transformed with pON177 sporulation plasmid (mat1-M mating cassette marked with *ura4*) (gift from Olaf Nielsen). Cells were harvested by centrifugation (2,500 rpm, 3 mins) and resuspended in 20 μl water. 20 μg boiled salmon sperm DNA, 1.5 μg pON177 DNA and 200 μl PEG-LiAC-TE (40% (w/v) PEG4000, 0.1 M LiAc, 1 mM EDTA, 10 mM TRIS at pH 7.5) were added then cells were incubated at 25°C for 3 days. Cells were harvested by centrifugation (2,500 rpm, 3 mins), washed and then transformants selected on EMM agar + 250 mg/L leucine. To induce sporulation, transformants were inoculated into EMM (without NH_4_Cl) + 1 mg/ml glutamic acid + 250 mg/L adenine + 250 mg/L leucine and incubated at 25°C for 3 days. Spores were harvested by treatment with 1:50 β-glucuronidase from *Helix pomatia* and stored in water at 4°C.

For visual screening, spores were resuspended in YE4S and incubated at 32°C for 22–24 hours. 5 μg/ml DiOC_6_ was added to 50 μl of cells then incubated at room temperature for 1 minute before visualisation using a Zeiss Axioskop 40 microscope with a 63X, 1.4 NA objective. 152 potential aberrant N/C ratio mutants were identified and subject to a secondary round of screening as described for the primary screen. The previously described 5300 control strain in which the KanR deletion cassette is inserted in pseudogene SPAC212.05c was used as a control for this and subsequent screening stages [34]. 60 potential N/C aberrant ratio mutants, identified in both primary and secondary screens, were carried forward for tertiary screening.

For tertiary screening, heterozygous diploid gene deletions were first transformed with a NE marker construct designed to integrate into the KanMX4 deletion cassette. NE protein Ish1 was tagged with yEGFP (marked with HygR selection marker with pAgTEF promoter and tCYC1 terminator) at its native locus in wild type cells using the pYM25 plasmid and the PCR-based transformation method previously described [35]. gDNA was isolated from transformants using the MasterPure Yeast DNA Purification Kit (Epicentre) and manufacturer’s instructions and a DNA fragment (Fragment A) with the *ish1* open reading frame (ORF) and 1 kb upstream sequence, yEGFP and HygR marker was amplified by PCR. Two synthetic DNA fragments, each with 400 bp homology to KanMX4 (Integrated DNA technologies (gBlocks)), with Fragment A between them, were cloned into a linearised pUC19 vector backbone using a Gibson Assembly Cloning Kit (NEB). The plasmid was digested with KpnI-HF (NEB) and SphI-HF (NEB) to yield the NE marker construct which was transformed into heterozygous deletion mutants as described for pON177 transformation above. Integration of the NE marker construct into the KanMX4 deletion cassette was confirmed by colony PCR and by microscopy; heterozygous diploids were sporulated and asci confirmed to contain two spores containing Ish1-yEGFP and two wild type spores. We confirmed that the N/C ratio of cells with Ish1-yEGFP integrated at the native *ish1* locus is not significantly different from that of cells containing the alternative NE marker Cut11-GFP integrated at its native locus (Fig 1D and 1E).

Transformation with pON177, sporulation and harvesting of spores was carried out as described for the primary screen. As in the primary screen, spores were resuspended in YE4S and incubated at 32°C for 22–24 hours then imaged. Cells were imaged using a DeltaVision Elite microscope (Applied Precision). N/C ratio of each strain was measured.

23 mutants with a mean N/C ratio more than one standard deviation greater than or smaller than the wild type population mean and significantly different from the wild type population mean with a p value ≤0.002, and two further mutants with a mean N/C ratio within one standard deviation of the wild type mean but significantly different from the wild type population mean with a p value <0.0001, were identified as aberrant N/C ratio candidates. The gene deletions in these 25 N/C ratio mutants were confirmed by colony PCR using CP3 and CP5 gene specific primers [18] with primers internal to the NE marker construct.

The haploid wild type control used for this screen was derived from the previously described 5300 heterozygous diploid control strain in which the KanR deletion cassette is inserted in pseudogene SPAC212.05c [34]. It is of note that this wild type control strain, in which the Ish1-yEGFP construct is integrated into the deletion cassette deleting the 5300 pseudogene, displayed an N/C ratio of 0.05 in this screen, with other strains distributed around this value (Fig 2A). When Ish1-yEGFP integrated at the native *ish1* locus was used to mark the nuclear envelope of wild type haploid cells, an N/C ratio of 0.08 was observed (Fig 1D and 1E). As the native *ish1* locus is undisrupted in the screened strains, the introduction of an additional copy of *ish1*, which encodes a nuclear envelope protein, may cause the observed wild type N/C ratio reduction from 0.08 to 0.05.

### Imaging and image analysis

Fluorescence imaging was carried out using a DeltaVision Elite microscope (Applied Precision) comprised of an Olympus IX71 wide-field inverted fluorescence microscope, an Olympus Plan APO 60X, 1.4 NA oil objective and a Photometrics CoolSNAP HQ2 camera (Roper Scientific) in an IMSOL ‘imcubator’ Environment Control System unless otherwise stated. Imaging was carried out in liquid media on glass slides at 25°C unless otherwise stated. Images were acquired in 0.2 μm or 0.4 μm z-sections over 4.4 μm, with a brightfield reference image in the middle of the sample, and deconvolved using SoftWorx (Applied Precision) unless otherwise stated. Representative images shown are maximum intensity projections of deconvolved images unless otherwise stated. For FM 4–64 staining experiments, fluorescence imaging was carried out using a wide-field inverted microscope comprised of a Nikon Eclipse Ti2 base, a Nikon Plan Apo 100X, 1.45 NA oil objective and a Photometrics Prime camera and images were deconvolved using Huygens. Colonies growing on solid agar plates were imaged using a Zeiss Axioskop 40 microscope with 10X Nikon objective, 2.5X Zeiss Optovar and Sony Alpha NEX-5 camera.

### FM 4–64 staining

For vacuole staining, cells were grown in the presence of 50 μM FM 4–64 (Molecular Probes) for 30 minutes at 32°C, then washed in YE4S and incubated in YE4S at 32°C for 35 minutes before imaging.

### Nuclear volume, cell volume and N/C ratio determination

To determine nuclear volume, cell volume and N/C ratio, cells and nuclei were manually measured, in brightfield reference images and fluorescence images of a NE marker respectively, using image J (NIH) as described previously [7]. n ≥ 50 cells per strain or condition.

### Bioinformatic analysis and statistical tests

Unless otherwise indicated, two-tailed unpaired student’s t-tests were used to determine significance of difference between two populations of N/C ratio measurements. Welch’s correction was used where indicated when an F test indicated that the variances of the two populations were significantly different.

Gene list enrichment analysis was carried out using GO::TermFinder [23] with PomBase annotations [24] and default parameters. Network analysis was carried out using esyN software [25] with PomBase curated interactions [24] and BioGRID curated interactions [36]. Default parameters, with “High and Low” setting for BioGRID interactions selected, were used.

## Supporting information

S1 Appendix Representative images of aberrant N/C ratio mutants identified by genetic screen. Representative images of wild type control strain and strains carrying a deletion in the gene indicated. Strains are categorised into wild type, low N/C ratio mutants and high N/C ratio mutants. Within these categories strains are in order of increasing N/C ratio. Brightfield (magenta), ish1-yEGFP (yellow). Cells without Ish1-yEGFP do not contain the gene deletion so were not assessed. Scale bars: 10 μm.
(PNG)

S1 Table N/C ratios and product functions of genes deleted in strains screened by imaging NE marker. ^a^Code and name of deleted gene. U: unassigned.
^b^Mean, standard deviation (SD) and coefficient of variation (COV) of N/C ratio measurements for each strain (n = 50 cells per strain).
^c^Significance (p value) of difference between each population and wild type (WT) population determined by student’s t-test with Welch’s correction.
^d^Function of product of gene [24].
• Nuclear size mutant.
(DOCX)

S2 Table Statistical analysis of N/C ratios of cells with cellular volumes within two standard deviations of the wild type mean for N/C ratio mutants. For each N/C ratio mutant, n value (number of values), mean and standard deviation (SD) and results of two-tailed Mann-Whitney U test (used as D’Agostino and Pearson test indicated some populations were not normal) to determine significance of difference from wild type population shown.
(DOCX)

S3 Table *S*. *pombe* strains used in this study. Genotype and origin of *S*. *pombe* strains used in this study.
(DOCX)

S4 Table Raw data values for screen measurements. Cell length, cell width, cell volume, nucleus volume and N/C ratio measurements for individual cells in populations of 60 mutants and wild type control screened by imaging NE marker and described in Table 1, S1 Table and Fig 2A.
(XLSX)

S5 Table Raw data values for Fig 1E and Fig 6A. N/C ratio measurements for individual cells in populations described in Fig 1E and Fig 6A.
(XLSX)

S1 Fig Relationships between cellular volume and nuclear volume and N/C ratio. (A) Mean nuclear volume plotted against mean cellular volume for each of the 60 strains imaged with an integrated nuclear envelope marker and wild type cells (n = 50 cells per strain). High (green) and low (blue) N/C ratio mutants and wild type control strain (red) coloured.
(B) Mean N/C ratio plotted against mean cellular volume for each of the 60 strains imaged with nuclear envelope marker and wild type cells (n = 50 cells per strain). High (green) and low (blue) N/C ratio mutants and wild type control strain (red) coloured.
(C) Gradient of the regression line between cellular volume and N/C ratio within population of 50 cells for each of 17 high N/C ratio (green), low N/C ratio (blue) and wild type control strain (red) cells plotted against mean N/C ratio of each strain.
(TIF)

S2 Fig Vacuole distribution is not dramatically altered in extreme N/C ratio mutants. Representative images of vacuole staining dye FM 4–64 (magenta) and Ish1-yeGFP (green) for wild type (WT) cells and strains carrying deletions in the genes indicated. The indicated strains are the four most extreme low N/C ratio and four most extreme high N/C ratio deletion mutants identified by genetic screening. Cells without Ish1-yEGFP do not contain the gene deletion so were not assessed. Scale bar: 5 μm.
(TIF)
