# Supplementary figures and images for: A systematic genetic screen identifies essential factors involved in nuclear size control
Source: PLoS Genet. 2019 Feb 13;15(2):e1007929. doi: 10.1371/journal.pgen.1007929 (PMC6391033; doi:10.1371/journal.pgen.1007929)

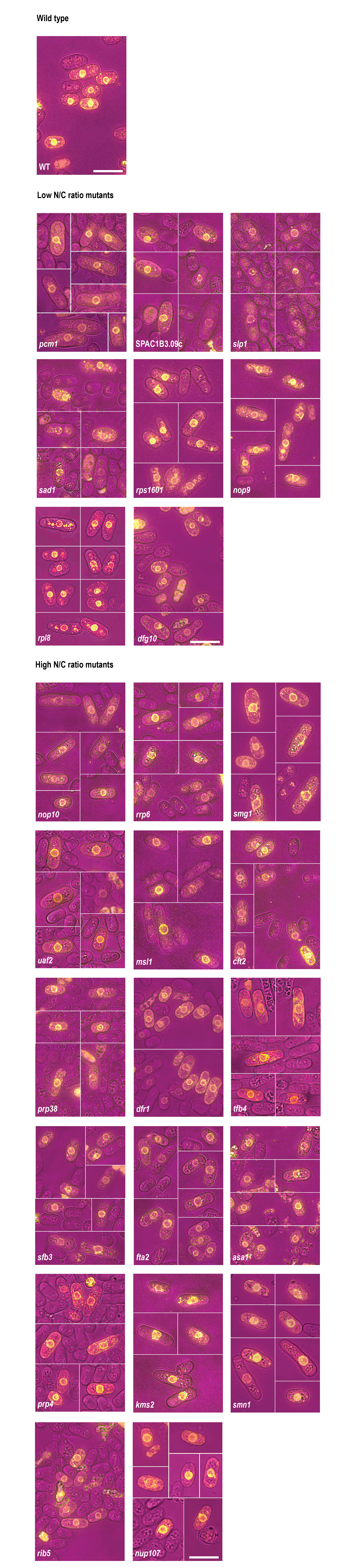

Supplement: S1 Appendix — Representative images of wild type control strain and strains carrying a deletion in the gene indicated. Strains are categorised into wild type, low N/C ratio mutants and high N/C ratio mutants. Within these categories strains are in order of increasing N/C ratio. Brightfield (magenta), ish1-yEGFP (yellow). Cells without Ish1-yEGFP do not contain the gene deletion so were not assessed. Scale bars: 10 μm. (PNG) [file pgen.1007929.s001.png]

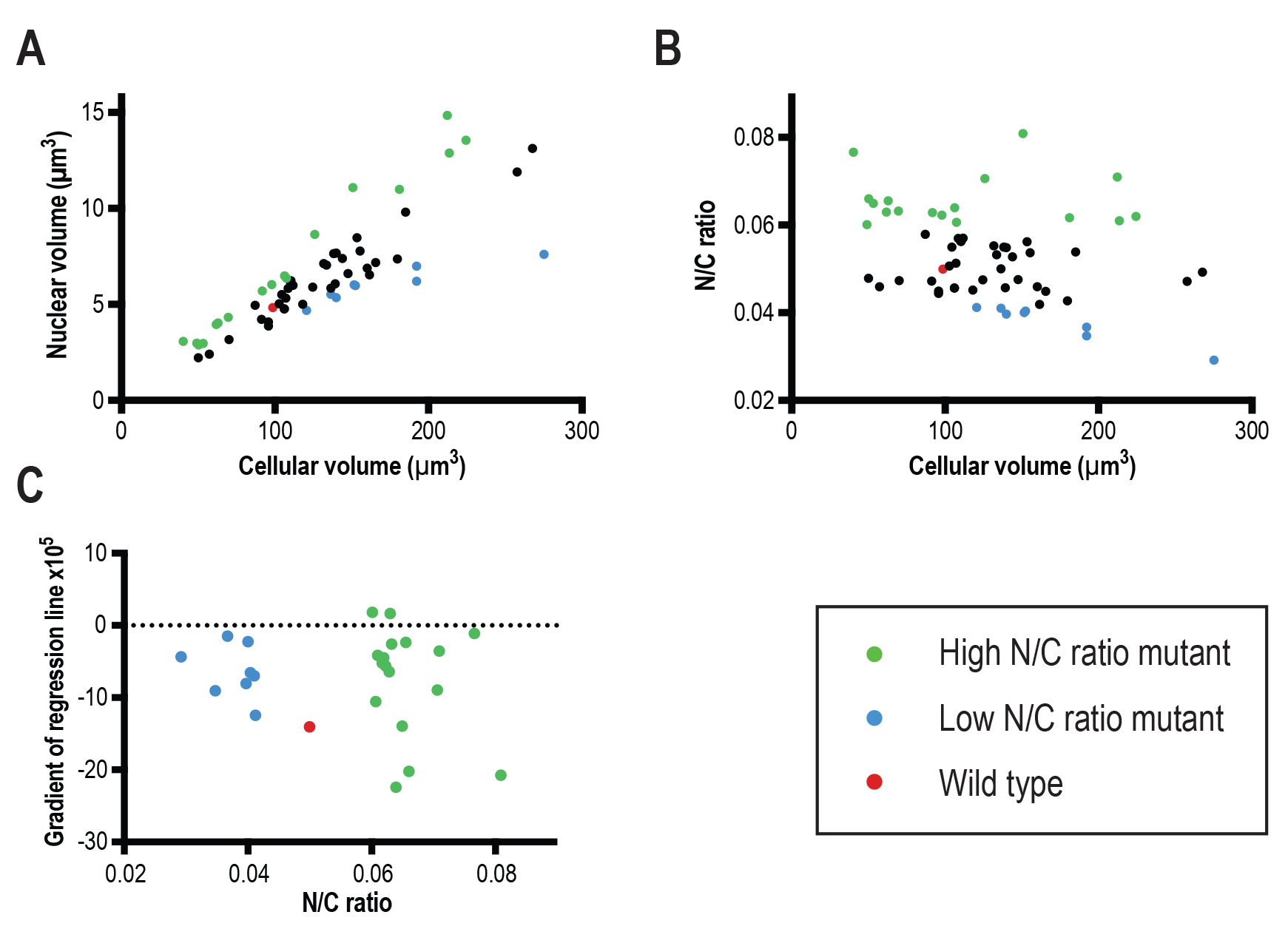

Supplement: S1 Fig — (A) Mean nuclear volume plotted against mean cellular volume for each of the 60 strains imaged with an integrated nuclear envelope marker and wild type cells (n = 50 cells per strain). High (green) and low (blue) N/C ratio mutants and wild type control strain (red) coloured. (B) Mean N/C ratio plotted against mean cellular volume for each of the 60 strains imaged with nuclear envelope marker and wild type cells (n = 50 cells per strain). High (green) and low (blue) N/C ratio mutants and wild type control strain (red) coloured. (C) Gradient of the regression line between cellular volume and N/C ratio within population of 50 cells for each of 17 high N/C ratio (green), low N/C ratio (blue) and wild type control strain (red) cells plotted against mean N/C ratio of each strain. (TIF) [file pgen.1007929.s007.tif]

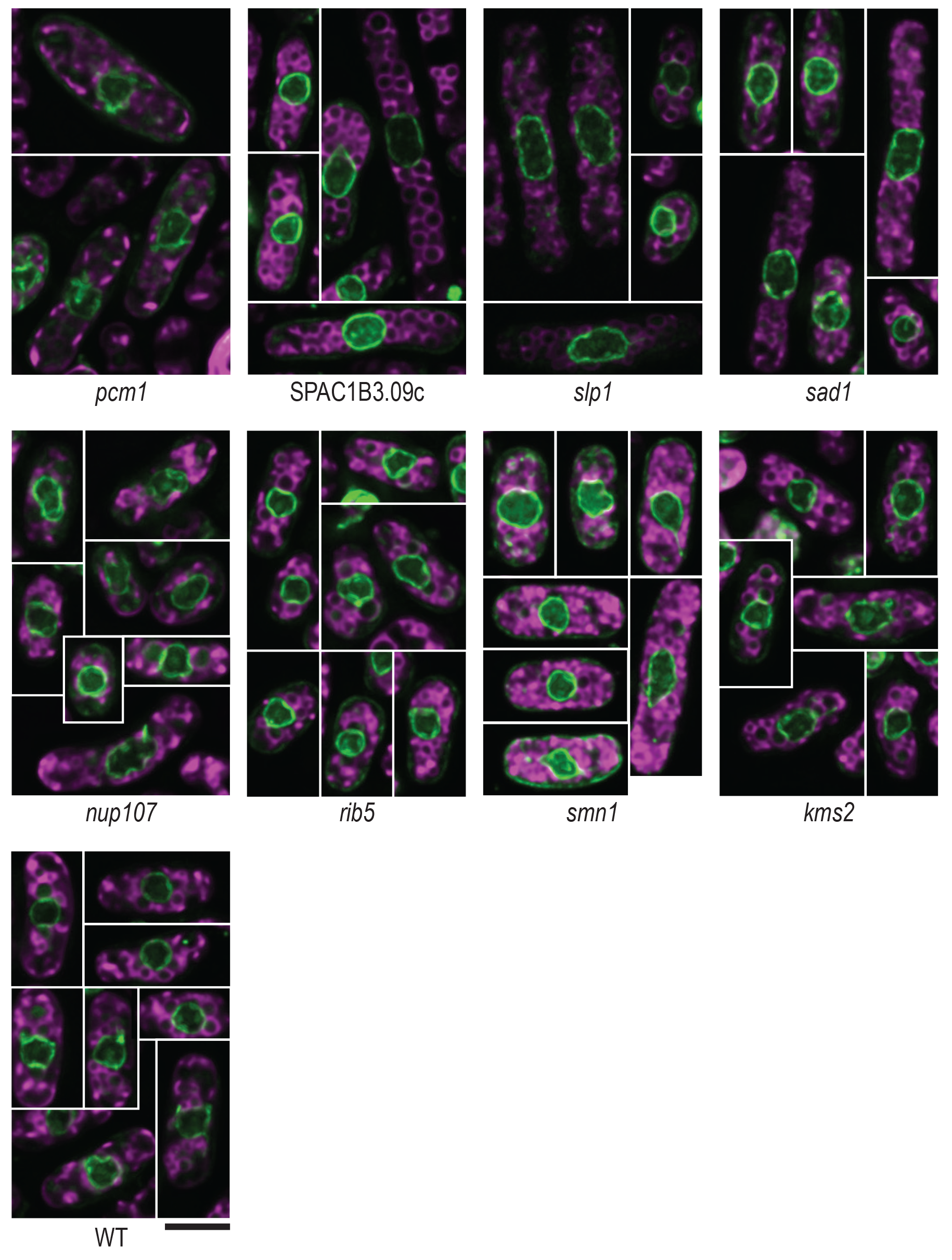

Supplement: S2 Fig — Representative images of vacuole staining dye FM 4–64 (magenta) and Ish1-yeGFP (green) for wild type (WT) cells and strains carrying deletions in the genes indicated. The indicated strains are the four most extreme low N/C ratio and four most extreme high N/C ratio deletion mutants identified by genetic screening. Cells without Ish1-yEGFP do not contain the gene deletion so were not assessed. Scale bar: 5 μm. (TIF) [file pgen.1007929.s008.tif]
