# Supplementary material for: A systematic genetic screen identifies essential factors involved in nuclear size control
Source: PLoS Genet. 2019 Feb 13;15(2):e1007929. doi: 10.1371/journal.pgen.1007929 (PMC6391033; doi:10.1371/journal.pgen.1007929)
Supplement: S1 Table — aCode and name of deleted gene. U: unassigned. bMean, standard deviation (SD) and coefficient of variation (COV) of N/C ratio measurements for each strain (n = 50 cells per strain). cSignificance (p value) of difference between each population and wild type (WT) population determined by student’s t-test with Welch’s correction. dFunction of product of gene [24]. • Nuclear size mutant. (DOCX) [file pgen.1007929.s002.docx]

| **Gene deleted^a^** | | **N/C ratio^b^** | | | **p value^c^** | **Function^d^** |
| --- | --- | --- | --- | --- | --- | --- |
| **Code** | **Name** | **Mean** | **SD** | **COV** |  |  |
| • SPCC330.10 | *pcm1* | 0.0292 | 0.0085 | 0.2928 | <0.0001 | P-TEFb-cap methyltransferase Pcm1 |
| • SPAC1B3.09c | U | 0.0361 | 0.0136 | 0.3772 | <0.0001 | Noc2p-Noc3p complex subunit Noc2 family (predicted) |
| • SPAC821.08c | *slp1* | 0.0367 | 0.0076 | 0.2078 | <0.0001 | substrate-specific mitotic metaphase/anaphase APC coactivator Slp1 |
| • SPBC12D12.01 | *sad1* | 0.0397 | 0.0102 | 0.2570 | <0.0001 | spindle pole body SUN domain protein Sad1 |
| • SPBC18H10.14 | *rps1601* | 0.0400 | 0.0090 | 0.2238 | <0.0001 | 40S ribosomal protein S16 (predicted) |
| • SPAC6G9.02c | *nop9* | 0.0404 | 0.0097 | 0.2393 | <0.0001 | pumilio family RNA-binding protein Nop9 (predicted) |
| • SPBC29A3.04 | *rpl8* | 0.0410 | 0.0088 | 0.2134 | <0.0001 | 60S ribosomal protein L7a/L8 (predicted) |
| • SPAC7D4.09c | *dfg10* | 0.0412 | 0.0099 | 0.2393 | <0.0001 | 3-oxo-5-alpha-steroid 4-dehydrogenase (predicted) |
| SPAC1834.06c | *pmo25* | 0.0419 | 0.0124 | 0.2956 | 0.0004 | mo25 family protein Pmo25 |
| SPBC18H10.12c | *rpl701* | 0.0427 | 0.0137 | 0.3212 | 0.0027 | 60S ribosomal protein L7a involved in ribosome biogenesis |
| SPAC11E3.02c | U | 0.0444 | 0.0133 | 0.3002 | 0.0174 | C2 domain protein |
| SPAC2F3.03c | *rpa49* | 0.0449 | 0.0120 | 0.2668 | 0.0188 | DNA-directed RNA polymerase I complex subunit Rpa49 (predicted) |
| SPCC576.05 | *sac3* | 0.0450 | 0.0132 | 0.2933 | 0.0305 | nuclear export factor Sac3 (predicted) |
| SPBC337.12 | *red5* | 0.0452 | 0.0120 | 0.2651 | 0.0267 | human ZC3H3 homolog |
| SPBC19F8.07 | *mcs6* | 0.0456 | 0.0123 | 0.2687 | 0.0480 | TFIIH associated cyclin-dependent protein kinase Mcs6 |
| SPBC336.08 | *spc24* | 0.0457 | 0.0141 | 0.3089 | 0.0764 | NMS complex subunit Spc24 |
| SPAC13A11.02c | *erg11* | 0.0459 | 0.0129 | 0.2820 | 0.0751 | sterol 14-demethylase Erg11 (predicted) |
| SPAC19A8.13 | *usp101* | 0.0460 | 0.0148 | 0.3220 | 0.1076 | U1 snRNP-associated protein Usp101 |
| SPBC11C11.03 | *ndc80* | 0.0471 | 0.0232 | 0.4922 | 0.4267 | NMS complex subunit Ndc80 |
| SPBC21H7.02 | *taf10* | 0.0472 | 0.0187 | 0.3968 | 0.3597 | SAGA complex/transcription factor TFIID complex subunit Taf10 |
| SPBC1105.07c | *pci2* | 0.0473 | 0.0089 | 0.1884 | 0.1472 | TREX complex subunit Pci2 (predicted) |
| SPAPB1E7.01c | U | 0.0475 | 0.0114 | 0.2394 | 0.2358 | conserved fungal family |
| SPBC146.07 | *prp2* | 0.0476 | 0.0199 | 0.4194 | 0.4426 | U2AF large subunit (U2AF-59) |
| SPCC63.10c | *sec59* | 0.0478 | 0.0108 | 0.2256 | 0.2938 | dolichol kinase Sec59 (predicted) |
| SPBC12D12.08c | *ned8* | 0.0493 | 0.0093 | 0.1886 | 0.7074 | ubiquitin-like protein modifier for cullin Ned8 |
| WT | N/A | 0.0500 | 0.0092 | 0.1834 | N/A | N/A |
| SPBC21.01 | *mis17* | 0.0500 | 0.0172 | 0.3434 | 0.9765 | kinetochore protein, CENP-U ortholog Mis17 |
| SPBC336.07 | *sfc3* | 0.0507 | 0.0174 | 0.3438 | 0.8009 | transcription factor TFIIIC complex B box binding subunit Sfc3 |
| SPAC110.04c | *pss1* | 0.0513 | 0.0128 | 0.2499 | 0.5468 | heat shock protein Pss1 |
| SPBC1271.04c | *dys1* | 0.0518 | 0.0146 | 0.2815 | 0.4479 | eIF-5A-deoxyhypusine synthase Dys1 (predicted) |
| SPBP35G2.09 | *usp103* | 0.0528 | 0.0103 | 0.1958 | 0.1521 | U1 snRNP-associated protein Usp103 |
| SPCC757.10 | *vph2* | 0.0532 | 0.0116 | 0.2174 | 0.1243 | endoplasmic reticulum membrane protein involved in assembly of the V-ATPase (predicted) |
| SPBC29A10.15 | *orc1* | 0.0537 | 0.0145 | 0.2705 | 0.1300 | origin recognition complex subunit Orc1 |
| SPCC1259.06 | *taf8* | 0.0539 | 0.0108 | 0.2012 | 0.0545 | transcription factor TFIID complex subunit 8 (predicted) |
| SPAC1250.01 | *snf21* | 0.0549 | 0.0150 | 0.2731 | 0.0508 | ATP-dependent DNA helicase Snf21 |
| SPAC222.11 | *hem13* | 0.0550 | 0.0127 | 0.2312 | 0.0266 | coproporphyrinogen III oxidase Hem13 (predicted) |
| SPBC27B12.02 | *mis19* | 0.0550 | 0.0151 | 0.2752 | 0.0475 | centromere protein Mis19/Eic1 |
| SPAC6F12.15c | *cut9* | 0.0553 | 0.0130 | 0.2354 | 0.0204 | anaphase-promoting complex, TPR lobe subcomplex subunit Cut9/Apc6 |
| SPBC1734.03 | *fol1* | 0.0560 | 0.0113 | 0.2013 | 0.0041 | trifunctional dihydropteroatesynthase/2-amino-4-hydroxy-6-hydro xymethyldihydropteridinediphosphokinase/ dihydroneopterin aldolase Fol1 (predicted) |
| SPBC14F5.08 | *med7* | 0.0562 | 0.0161 | 0.2871 | 0.0196 | mediator complex subunit Med7 |
| SPAC29E6.04 | *nnf1* | 0.0563 | 0.0154 | 0.2741 | 0.0149 | NMS complex subunit Nnf1 |
| SPAC12G12.05c | *taf9* | 0.0570 | 0.0197 | 0.3455 | 0.0255 | SAGA complex/ transcription initiation factor Taf9 |
| SPAC18B11.06 | *lcp5* | 0.0570 | 0.0142 | 0.2497 | 0.0042 | U3 snoRNP-associated protein Lcp5 (predicted) |
| SPAC6F12.11c | *sfc1* | 0.0579 | 0.0109 | 0.1885 | 0.0002 | transcription factor TFIIIC complex A box associated subunit Sfc1 |
| • SPAP27G11.13c | *nop10* | 0.0601 | 0.0151 | 0.2507 | 0.0001 | snoRNP pseudouridylase box H/ACA snoRNP complex protein (predicted) |
| • SPAC1F3.01 | *rrp6* | 0.0607 | 0.0213 | 0.3508 | 0.0017 | exosome 3'-5' exoribonuclease subunit Rrp6 (predicted) |
| • SPBC4B4.05 | *smg1* | 0.0610 | 0.0180 | 0.2951 | 0.0002 | Sm snRNP core protein Smg1 |
| • SPAP8A3.06 | *uaf2* | 0.0617 | 0.0122 | 0.1983 | <0.0001 | U2AF small subunit, U2AF-23 |
| • SPBC8D2.09c | *msl1* | 0.0620 | 0.0161 | 0.2595 | <0.0001 | U2 snRNP-associated protein Msl1 (predicted) |
| • SPBC1709.15c | *cft2* | 0.0622 | 0.0180 | 0.2889 | <0.0001 | cleavage factor two Cft2/polyadenylation factor CPSF-73 (predicted) |
| • SPBC19C2.08 | *prp38* | 0.0628 | 0.0171 | 0.2717 | <0.0001 | U4/U6 x U5 tri-snRNP complex subunit Prp38 |
| • SPCC1223.08c | *dfr1* | 0.0630 | 0.0132 | 0.2096 | <0.0001 | dihydrofolate reductase/ serine hydrolase family fusion protein Dfr1 |
| • SPBC30B4.07c | *tfb4* | 0.0632 | 0.0147 | 0.2323 | <0.0001 | transcription factor TFIIH complex subunit Tfb4 |
| • SPBC4.03c | *sfb3* | 0.0639 | 0.0148 | 0.2309 | <0.0001 | COPII-coated vesicle component Sfb3 (predicted) |
| • SPAC1783.03 | *fta2* | 0.0649 | 0.0239 | 0.3684 | 0.0001 | kinetochore protein, CENP-P ortholog Fta2 |
| • SPCC777.14 | *prp4* | 0.0655 | 0.0150 | 0.2295 | <0.0001 | serine/threonine protein kinase Prp4 |
| • SPAC1006.02 | *asa1* | 0.0660 | 0.0216 | 0.3267 | <0.0001 | Astra associated protein 1 Asa1 |
| • SPBC947.12 | *kms2* | 0.0706 | 0.0204 | 0.2883 | <0.0001 | spindle pole body protein Kms2 |
| • SPAC2G11.08c | *smn1* | 0.0709 | 0.0185 | 0.2613 | <0.0001 | SMN family protein Smn1 |
| • SPCC1450.13c | *rib5* | 0.0766 | 0.0170 | 0.2221 | <0.0001 | riboflavin synthase Rib5 |
| • SPBC428.01c | *nup107* | 0.0809 | 0.0319 | 0.3945 | <0.0001 | nucleoporin Nup107 |
