# Supplementary material for: A systematic genetic screen identifies essential factors involved in nuclear size control
Source: PLoS Genet. 2019 Feb 13;15(2):e1007929. doi: 10.1371/journal.pgen.1007929 (PMC6391033; doi:10.1371/journal.pgen.1007929)
Supplement: S2 Table — For each N/C ratio mutant, n value (number of values), mean and standard deviation (SD) and results of two-tailed Mann-Whitney U test (used as D’Agostino and Pearson test indicated some populations were not normal) to determine significance of difference from wild type population shown. (DOCX) [file pgen.1007929.s003.docx]

| **Strain** | **Number of values** | **Mean** | **SD** | **Mann-Whitney U test against WT** | | |
| --- | --- | --- | --- | --- | --- | --- |
|  |  |  |  | **p value** | **p < 0.05?** | **Mann-Whitney U** |
| *pcm1* | 2 | 0.036 | 0.004 | 0.0261 | Yes | 6 |
| SPAC1B3.09c | 16 | 0.044 | 0.016 | 0.1162 | No | 282 |
| *slp1* | 14 | 0.039 | 0.0076 | 0.0001 | Yes | 120 |
| *sad1* | 24 | 0.042 | 0.011 | 0.0024 | Yes | 325 |
| *rps1601* | 27 | 0.042 | 0.0086 | 0.0001 | Yes | 312 |
| *nop9* | 28 | 0.043 | 0.01 | 0.0099 | Yes | 434 |
| *rpl8* | 34 | 0.042 | 0.0087 | 0.0001 | Yes | 415 |
| *dfg10* | 40 | 0.044 | 0.0088 | 0.0028 | Yes | 607 |
| *nop10* | 19 | 0.065 | 0.015 | <0.0001 | Yes | 171 |
| *rrp6* | 40 | 0.06 | 0.02 | 0.0166 | Yes | 675 |
| *smg1* | 7 | 0.067 | 0.017 | 0.0057 | Yes | 62 |
| *uaf2* | 16 | 0.066 | 0.013 | <0.0001 | Yes | 117 |
| *msl1* | 6 | 0.061 | 0.014 | 0.0621 | No | 76 |
| *cft2* | 45 | 0.063 | 0.018 | <0.0001 | Yes | 538 |
| *prp38* | 47 | 0.063 | 0.017 | <0.0001 | Yes | 611 |
| *dfr1* | 13 | 0.057 | 0.015 | 0.0796 | No | 212 |
| *tfb4* | 12 | 0.058 | 0.017 | 0.055 | No | 184 |
| *sfb3* | 43 | 0.065 | 0.014 | <0.0001 | Yes | 360 |
| *fta2* | 12 | 0.065 | 0.03 | 0.0481 | Yes | 181 |
| *prp4* | 17 | 0.067 | 0.015 | <0.0001 | Yes | 133 |
| *asa1* | 19 | 0.061 | 0.016 | 0.0038 | Yes | 251 |
| *kms2* | 38 | 0.072 | 0.022 | <0.0001 | Yes | 263 |
| *smn1* | 14 | 0.072 | 0.014 | <0.0001 | Yes | 58 |
| *rib5* | 17 | 0.077 | 0.016 | <0.0001 | Yes | 34 |
| *nup107* | 30 | 0.087 | 0.032 | <0.0001 | Yes | 149 |
| WT | 48 | 0.05 | 0.009 | N/A | N/A | N/A |
