# Supplementary material for: A systematic genetic screen identifies essential factors involved in nuclear size control
Source: PLoS Genet. 2019 Feb 13;15(2):e1007929. doi: 10.1371/journal.pgen.1007929 (PMC6391033; doi:10.1371/journal.pgen.1007929)
Supplement: S3 Table — Genotype and origin of S. pombe strains used in this study. (DOCX) [file pgen.1007929.s004.docx]

| **Strain** | **Genotype** | **Origin** |
| --- | --- | --- |
| PN3779 | *cut11-GFP::ura4+ leu1-32 ura4-D18 h+* | Lab collection |
| HC58 | *ish1-yEGFP::hphNT1 h?* | This study |
| HC180 | *pcm1∆::ish1-yEGFP-hphNT1/pcm1+, ade6-M210/ade6-M216, leu1-32/leu1-32, ura4-D18/ura4-D18, h+/h+* | This study |
| HC181 | *SPAC1B3.09c∆::ish1-yEGFP-hphNT1/SPAC1B3.09c+, ade6-M210/ade6-M216, leu1-32/leu1-32, ura4-D18/ura4-D18, h+/h+* | This study |
| HC182 | *slp1∆::ish1-yEGFP-hphNT1/slp1+, ade6-M210/ade6-M216, leu1-32/leu1-32, ura4-D18/ura4-D18, h+/h+* | This study |
| HC183 | *sad1∆::ish1-yEGFP-hphNT1/sad1+, ade6-M210/ade6-M216, leu1-32/leu1-32, ura4-D18/ura4-D18, h+/h+* | This study |
| HC184 | *rps1601∆::ish1-yEGFP-hphNT1/rps1601+, ade6-M210/ade6-M216, leu1-32/leu1-32, ura4-D18/ura4-D18, h+/h+* | This study |
| HC185 | *nop9∆::ish1-yEGFP-hphNT1/nop9+, ade6-M210/ade6-M216, leu1-32/leu1-32, ura4-D18/ura4-D18, h+/h+* | This study |
| HC186 | *rpl8∆::ish1-yEGFP-hphNT1/rpl8+, ade6-M210/ade6-M216, leu1-32/leu1-32, ura4-D18/ura4-D18, h+/h+* | This study |
| HC187 | *dfg10∆::ish1-yEGFP-hphNT1/dfg10+, ade6-M210/ade6-M216, leu1-32/leu1-32, ura4-D18/ura4-D18, h+/h+* | This study |
| HC188 | *pmo25∆::ish1-yEGFP-hphNT1/pmo25+, ade6-M210/ade6-M216, leu1-32/leu1-32, ura4-D18/ura4-D18, h+/h+* | This study |
| HC189 | *rpl701∆::ish1-yEGFP-hphNT1/rpl701+, ade6-M210/ade6-M216, leu1-32/leu1-32, ura4-D18/ura4-D18, h+/h+* | This study |
| HC190 | *SPAC11E3.02c∆::ish1-yEGFP-hphNT1/SPAC11E3.02c+, ade6-M210/ade6-M216, leu1-32/leu1-32, ura4-D18/ura4-D18, h+/h+* | This study |
| HC191 | *rpa49∆::ish1-yEGFP-hphNT1/rpa49+, ade6-M210/ade6-M216, leu1-32/leu1-32, ura4-D18/ura4-D18, h+/h+* | This study |
| HC192 | *sac3∆::ish1-yEGFP-hphNT1/sac3+, ade6-M210/ade6-M216, leu1-32/leu1-32, ura4-D18/ura4-D18, h+/h+* | This study |
| HC193 | *red5∆::ish1-yEGFP-hphNT1/red5+, ade6-M210/ade6-M216, leu1-32/leu1-32, ura4-D18/ura4-D18, h+/h+* | This study |
| HC194 | *mcs6∆::ish1-yEGFP-hphNT1/mcs6+, ade6-M210/ade6-M216, leu1-32/leu1-32, ura4-D18/ura4-D18, h+/h+* | This study |
| HC195 | *spc24∆::ish1-yEGFP-hphNT1/spc24+, ade6-M210/ade6-M216, leu1-32/leu1-32, ura4-D18/ura4-D18, h+/h+* | This study |
| HC196 | *erg11∆::ish1-yEGFP-hphNT1/erg11+, ade6-M210/ade6-M216, leu1-32/leu1-32, ura4-D18/ura4-D18, h+/h+* | This study |
| HC197 | *usp101∆::ish1-yEGFP-hphNT1/usp101+, ade6-M210/ade6-M216, leu1-32/leu1-32, ura4-D18/ura4-D18, h+/h+* | This study |
| HC198 | *ndc80∆::ish1-yEGFP-hphNT1/ndc80+, ade6-M210/ade6-M216, leu1-32/leu1-32, ura4-D18/ura4-D18, h+/h+* | This study |
| HC199 | *taf10∆::ish1-yEGFP-hphNT1/taf10+, ade6-M210/ade6-M216, leu1-32/leu1-32, ura4-D18/ura4-D18, h+/h+* | This study |
| HC200 | *pci2∆::ish1-yEGFP-hphNT1/pci2+, ade6-M210/ade6-M216, leu1-32/leu1-32, ura4-D18/ura4-D18, h+/h+* | This study |
| HC201 | *SPAPB1E7.01c∆::ish1-yEGFP-hphNT1/SPAPB1E7.01c+, ade6-M210/ade6-M216, leu1-32/leu1-32, ura4-D18/ura4-D18, h+/h+* | This study |
| HC202 | *prp2∆::ish1-yEGFP-hphNT1/prp2+, ade6-M210/ade6-M216, leu1-32/leu1-32, ura4-D18/ura4-D18, h+/h+* | This study |
| HC203 | *sec59∆::ish1-yEGFP-hphNT1/sec59+, ade6-M210/ade6-M216, leu1-32/leu1-32, ura4-D18/ura4-D18, h+/h+* | This study |
| HC204 | *ned8∆::ish1-yEGFP-hphNT1/ned8+, ade6-M210/ade6-M216, leu1-32/leu1-32, ura4-D18/ura4-D18, h+/h+* | This study |
| HC205 | *mis17∆::ish1-yEGFP-hphNT1/mis17+, ade6-M210/ade6-M216, leu1-32/leu1-32, ura4-D18/ura4-D18, h+/h+* | This study |
| HC206 | *sfc3∆::ish1-yEGFP-hphNT1/sfc3+, ade6-M210/ade6-M216, leu1-32/leu1-32, ura4-D18/ura4-D18, h+/h+* | This study |
| HC207 | *pss1∆::ish1-yEGFP-hphNT1/pss1+, ade6-M210/ade6-M216, leu1-32/leu1-32, ura4-D18/ura4-D18, h+/h+* | This study |
| HC208 | *dys1∆::ish1-yEGFP-hphNT1/dys1+, ade6-M210/ade6-M216, leu1-32/leu1-32, ura4-D18/ura4-D18, h+/h+* | This study |
| HC209 | *usp103∆::ish1-yEGFP-hphNT1/usp103+, ade6-M210/ade6-M216, leu1-32/leu1-32, ura4-D18/ura4-D18, h+/h+* | This study |
| HC210 | *vph2∆::ish1-yEGFP-hphNT1/vph2+, ade6-M210/ade6-M216, leu1-32/leu1-32, ura4-D18/ura4-D18, h+/h+* | This study |
| HC211 | *orc1∆::ish1-yEGFP-hphNT1/orc1+, ade6-M210/ade6-M216, leu1-32/leu1-32, ura4-D18/ura4-D18, h+/h+* | This study |
| HC212 | *taf8∆::ish1-yEGFP-hphNT1/taf8+, ade6-M210/ade6-M216, leu1-32/leu1-32, ura4-D18/ura4-D18, h+/h+* | This study |
| HC213 | *snf21∆::ish1-yEGFP-hphNT1/snf21+, ade6-M210/ade6-M216, leu1-32/leu1-32, ura4-D18/ura4-D18, h+/h+* | This study |
| HC214 | *hem13∆::ish1-yEGFP-hphNT1/hem13+, ade6-M210/ade6-M216, leu1-32/leu1-32, ura4-D18/ura4-D18, h+/h+* | This study |
| HC215 | *mis19∆::ish1-yEGFP-hphNT1/mis19+, ade6-M210/ade6-M216, leu1-32/leu1-32, ura4-D18/ura4-D18, h+/h+* | This study |
| HC216 | *cut9∆::ish1-yEGFP-hphNT1/cut9+, ade6-M210/ade6-M216, leu1-32/leu1-32, ura4-D18/ura4-D18, h+/h+* | This study |
| HC217 | *fol1∆::ish1-yEGFP-hphNT1/fol1+, ade6-M210/ade6-M216, leu1-32/leu1-32, ura4-D18/ura4-D18, h+/h+* | This study |
| HC218 | *med7∆::ish1-yEGFP-hphNT1/med7+, ade6-M210/ade6-M216, leu1-32/leu1-32, ura4-D18/ura4-D18, h+/h+* | This study |
| HC219 | *nnf1∆::ish1-yEGFP-hphNT1/nnf1+, ade6-M210/ade6-M216, leu1-32/leu1-32, ura4-D18/ura4-D18, h+/h+* | This study |
| HC220 | *taf9∆::ish1-yEGFP-hphNT1/taf9+, ade6-M210/ade6-M216, leu1-32/leu1-32, ura4-D18/ura4-D18, h+/h+* | This study |
| HC221 | *lcp5∆::ish1-yEGFP-hphNT1/lcp5+, ade6-M210/ade6-M216, leu1-32/leu1-32, ura4-D18/ura4-D18, h+/h+* | This study |
| HC222 | *sfc1∆::ish1-yEGFP-hphNT1/sfc1+, ade6-M210/ade6-M216, leu1-32/leu1-32, ura4-D18/ura4-D18, h+/h+* | This study |
| HC223 | *nop10∆::ish1-yEGFP-hphNT1/nop10+, ade6-M210/ade6-M216, leu1-32/leu1-32, ura4-D18/ura4-D18, h+/h+* | This study |
| HC224 | *rrp6∆::ish1-yEGFP-hphNT1/rrp6+, ade6-M210/ade6-M216, leu1-32/leu1-32, ura4-D18/ura4-D18, h+/h+* | This study |
| HC225 | *smg1∆::ish1-yEGFP-hphNT1/smg1+, ade6-M210/ade6-M216, leu1-32/leu1-32, ura4-D18/ura4-D18, h+/h+* | This study |
| HC226 | *uaf2∆::ish1-yEGFP-hphNT1/uaf2+, ade6-M210/ade6-M216, leu1-32/leu1-32, ura4-D18/ura4-D18, h+/h+* | This study |
| HC227 | *msl1∆::ish1-yEGFP-hphNT1/msl1+, ade6-M210/ade6-M216, leu1-32/leu1-32, ura4-D18/ura4-D18, h+/h+* | This study |
| HC228 | *cft2∆::ish1-yEGFP-hphNT1/cft2+, ade6-M210/ade6-M216, leu1-32/leu1-32, ura4-D18/ura4-D18, h+/h+* | This study |
| HC229 | *prp38∆::ish1-yEGFP-hphNT1/prp38+, ade6-M210/ade6-M216, leu1-32/leu1-32, ura4-D18/ura4-D18, h+/h+* | This study |
| HC230 | *dfr1∆::ish1-yEGFP-hphNT1/dfr1+, ade6-M210/ade6-M216, leu1-32/leu1-32, ura4-D18/ura4-D18, h+/h+* | This study |
| HC231 | *tfb4∆::ish1-yEGFP-hphNT1/tfb4+, ade6-M210/ade6-M216, leu1-32/leu1-32, ura4-D18/ura4-D18, h+/h+* | This study |
| HC232 | *sfb3∆::ish1-yEGFP-hphNT1/sfb3+, ade6-M210/ade6-M216, leu1-32/leu1-32, ura4-D18/ura4-D18, h+/h+* | This study |
| HC233 | *fta2∆::ish1-yEGFP-hphNT1/fta2+, ade6-M210/ade6-M216, leu1-32/leu1-32, ura4-D18/ura4-D18, h+/h+* | This study |
| HC234 | *prp4∆::ish1-yEGFP-hphNT1/prp4+, ade6-M210/ade6-M216, leu1-32/leu1-32, ura4-D18/ura4-D18, h+/h+* | This study |
| HC235 | *asa1∆::ish1-yEGFP-hphNT1/asa1+, ade6-M210/ade6-M216, leu1-32/leu1-32, ura4-D18/ura4-D18, h+/h+* | This study |
| HC236 | *kms2∆::ish1-yEGFP-hphNT1/kms2+, ade6-M210/ade6-M216, leu1-32/leu1-32, ura4-D18/ura4-D18, h+/h+* | This study |
| HC237 | *smn1∆::ish1-yEGFP-hphNT1/smn1+, ade6-M210/ade6-M216, leu1-32/leu1-32, ura4-D18/ura4-D18, h+/h+* | This study |
| HC238 | *rib5∆::ish1-yEGFP-hphNT1/rib5+, ade6-M210/ade6-M216, leu1-32/leu1-32, ura4-D18/ura4-D18, h+/h+* | This study |
| HC239 | *nup107∆::ish1-yEGFP-hphNT1/nup107+, ade6-M210/ade6-M216, leu1-32/leu1-32, ura4-D18/ura4-D18, h+/h+* | This study |
| HC240 | *SPAC212.05c∆::ish1-yEGFP-hphNT1/SPAC212.05c+, ade6-M210/ade6-M216, leu1-32/leu1-32, ura4-D18/ura4-D18, h+/h+* | This study |
| HC110 | *cdk9ΔC∷KanMX6 cut11-GFP::ura4+ ura4-D18 h?* | This study |
| HC111 | *cdk9^T212A^::KanMX6 cut11-GFP::ura4+ ura4-D18 h+* | This study |
